# A Case of Acute Budd-Chiari Syndrome Complicating Primary Antiphospholipid Syndrome Presenting as Acute Abdomen and Responding to Tight Anticoagulant Therapy

**DOI:** 10.1155/2016/9565427

**Published:** 2016-09-08

**Authors:** Naofumi Chinen, Yasushi Koyama, Shinji Sato, Yasuo Suzuki

**Affiliations:** Division of Rheumatology, Department of Internal Medicine, Tokai University School of Medicine, 142 Shimokasuya, Isehara 160-8582, Japan

## Abstract

A 34-year-old woman with primary antiphospholipid syndrome was admitted to the Gastroenterology Department of our hospital with fever, acute abdomen, watery diarrhea, and extremely high levels of inflammatory parameters. She had a history of left lower limb deep vein thrombosis and pulmonary embolism and was taking warfarin potassium. Acute gastroenteritis was suspected and an antibiotic was administered, but symptoms progressed. Abdominal ultrasonography showed occlusion of the left hepatic vein and the middle hepatic vein and her D-dimer level was high. Accordingly, Budd-Chiari syndrome was diagnosed and high-dose intravenous infusion of heparin was initiated. Her abdominal symptoms improved and the levels of inflammatory parameters and D-dimer decreased rapidly. It is known that antiphospholipid syndrome can be complicated by Budd-Chiari syndrome that usually occurs as subacute or chronic onset, but acute onset is rare. It is difficult to diagnose acute Budd-Chiari syndrome complicating antiphospholipid syndrome and this complication generally has a poor outcome. However, the present case can get early diagnosis and successful treatment with tight anticoagulant therapy.

## 1. Introduction

Antiphospholipid syndrome (APS) can cause arterial or venous thrombosis and various complications of pregnancy and is characterized by autoantibodies directed against phospholipids or circulating phospholipid-protein complexes. Abdominal symptoms rarely occur in APS and the causes include thrombosis at the hepatic vein, inferior vena cava, or portal vein and intestinal ischemia. Budd-Chiari syndrome (BCS) is a potentially life-threatening complication in APS [1]. BCS associated with APS is usually subacute or chronic, while acute BCS is rare and is reported to have a poor outcome [2]. We encountered an APS patient on warfarin therapy who was admitted with fever and acute abdomen. Acute BCS was suspected from the findings on enhanced computed tomography (CT) and hepatic Doppler ultrasonography along with elevation of a thrombotic marker. Early initiation of intravenous anticoagulant therapy achieved a good outcome. This is the suggestive case that tight anticoagulant therapy is useful in early-diagnosed acute BCS complicating APS.

## 2. Case Report

The patient was a 34-year-old woman. In 2012, she developed deep vein thrombosis of the left lower extremity and pulmonary embolism. Primary APS was diagnosed because she was positive for lupus anticoagulant, anticardiolipin antibody, and anticardiolipin-*β*2 GPI antibody. Subsequently, she was treated on anticoagulant therapy with warfarin. In 2015, she visited a local clinic with epigastric pain and watery diarrhea 3 days before admission to our hospital. Upper gastrointestinal endoscopy showed no abnormalities. However, her epigastric pain became worse, and fever (38°C), nausea, and vomiting occurred. Therefore, she presented to a local general hospital on the day before admission to our hospital. Since the cause of her symptoms was unclear, she was admitted to our hospital for investigation and treatment of fever and acute abdomen. On examination at admission, her blood pressure was 123/87 mmHg, pulse rate was 84/min, temperature was 38.4°C, and oxygen saturation was 94% (room air). There was no yellowing of the bulbar conjunctiva. Superficial lymph nodes were not palpable. Pulmonary sounds were clear and there was no heart murmur. The abdomen was distended and intestinal peristalsis was decreased. There was marked tenderness in the epigastric region and right hypochondrium but no muscular guarding. Edema of the left lower limb was observed.

Admission urinalysis showed no abnormal findings, while hematology tests revealed that the white blood cell count was 5.6 × 10^9^/L (88.0% neutrophils), hemoglobin was 121 g/L, hematocrit was 0.38/L, and platelet count was 175 × 10^9^/L. Regarding the coagulation profile, the prothrombin time international normalized ratio (PT-INR) was 2.44 and the activated partial thromboplastin time (APTT) ratio was 2.7, while D-dimer and fibrinogen markedly increased to 21.6 mg/L and 8.5 g/L, respectively. Biochemistry tests showed slight elevation of liver enzymes (aspartate transaminase, 65 IU/L; alanine transaminase, 51 IU/L; lactate dehydrogenase, 335 IU/L; alkaline phosphatase, 265 IU/L; and *γ*-glutamyl transpeptidase, 50 IU/L) and hypoalbuminemia (23 g/L), with no jaundice or kidney dysfunction. The serum level of C-reactive protein was markedly elevated (21.7 × 10^4^ *μ*g/L). Antinuclear antibody was positive at 1 : 320 (speckled pattern), anti-SS-A/Ro antibody was positive at 1 : 16, and rheumatoid factor was also positive, while anti-double-stranded DNA antibody, anti-Sm antibody, and myeloperoxidase antineutrophil cytoplasmic antibodies were negative. Regarding antiphospholipid antibodies, the lupus anticoagulant test (dilute Russell viper venom method) gave a result of 72.9 seconds and anti-cardiolipin-*β*2 GPI antibody was 90.5 U/mL, with both results being strongly positive. Blood and urine cultures were negative. Enhanced abdominal CT showed atrophy of the left lobe of the liver and a region of poor contrast enhancement.

**Figure 1:**
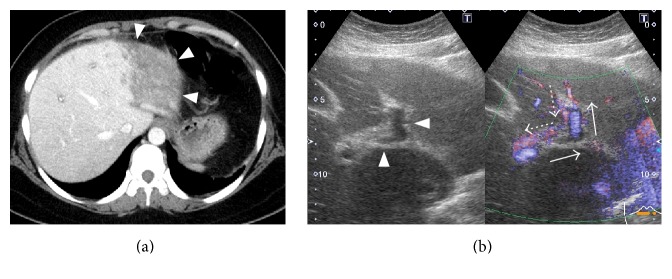
(a) Dynamic CT. There is poor enhancement of the lateral segment of the left lobe of the liver (arrowheads). (b) Doppler ultrasonography of the intrahepatic part of the portal vein. The left branch of the portal vein (arrowheads) shows reflux (arrows = normal blood flow; dotted arrows = blood flow in this patient).

**Figure 2:**
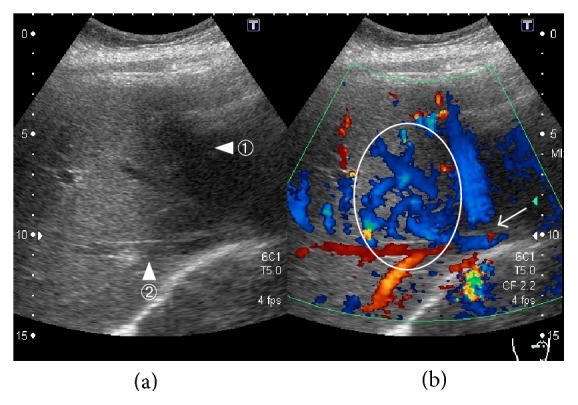
Doppler ultrasonography of the hepatic veins. (a) Middle hepatic vein (arrowhead ①) and right hepatic vein (arrowhead ②). (b) Blood flow is absent in parts of the middle hepatic vein (arrow) and increased blood flow is seen in the region from the middle hepatic vein to the right hepatic vein (circle).

**Figure 3:**
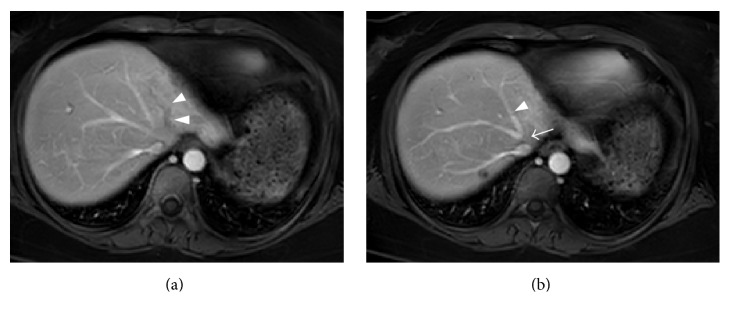
Contrast-enhanced MRI. (a) There is no contrast enhancement in the left hepatic vein (arrowhead). (b) The middle hepatic vein (arrowhead) is visualized, but there is no enhancement at its confluence with the inferior vena cava (arrow), suggesting occlusion.

**Figure 4:**
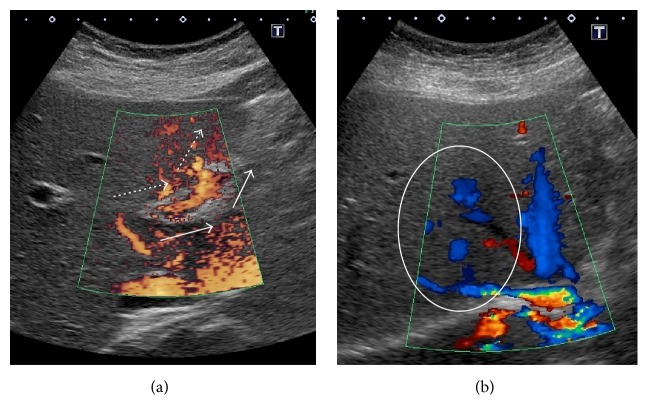
Doppler ultrasonography of the portal and hepatic veins after 5 months from discharge. (a) The reflux flow at the left branch of the portal vein had disappeared (arrows = normal blood flow; dotted arrows = blood flow in this patient). (b) The increased blood flow in the region from the middle hepatic vein to the right hepatic vein remained (circle).

Acute gastroenteritis was diagnosed and cefmetazole was started, while warfarin was continued. However, her abdominal pain did not improve and the antibiotic was switched to meropenem. Dynamic CT showed a small amount of ascites in the perihepatic region and expansion of the poorly enhanced area in the lateral segment of the left hepatic lobe, suggesting ischemia of the left lobe (Figure 1(a)). Since patient's condition was worsened under oral anticoagulation with warfarin and stopped oral intake because of strong abdominal pain, anticoagulant therapy was switched to heparin sodium (10,000 units/day) and further investigation was performed on the 4th day from admission. Doppler ultrasonography showed reflux of blood in the left branch of the portal vein (Figure 1(b)), as well as no blood flow in the left hepatic vein, obstruction in the central part of the middle hepatic vein, and increased blood flow in the right hepatic vein (Figure 2). Because of high levels of D-dimer and C-reactive protein and the Doppler imaging findings, thrombotic occlusion of the middle hepatic vein and left hepatic vein was strongly suspected, and acute BCS was diagnosed. On the 6th day, anticoagulant therapy was intensified, with infusion of heparin sodium in the range of 15000–25000 units/day to maintain an APTT ratio of 4.0–5.0 which was set at twice the baseline value. Subsequently, contrast-enhanced MRI showed no enhancement of the left hepatic vein and probable occlusion of the middle hepatic at its confluence with the inferior vena cava (Figure 3). After intensification of anticoagulant therapy, the patient's fever and abdominal pain resolved, and the levels of inflammatory parameters and D-dimer decreased without thrombocytopenia. On the 17th day, administration of warfarin was resumed and the PT-INR was maintained within the target range of 2.5–3.0; thereafter, heparin was discontinued on the 27th day. After discharge, there was no evidence of portal hypertension or ascites and the abnormal reflux flow at the left portal vein disappeared by abdominal ultrasonography 5 months later (Figure 4). She got good outcome under warfarin use alone.

## 3. Discussion

In Budd-Chiari syndrome (BCS), obstruction of the hepatic venous outflow tract occurs at a site from the small hepatic veins to the junction of the inferior vena cava with the right atrium, and this syndrome can have various causes [3]. BCS can be classified as primary or secondary; primary BCS is caused by venous thrombus or phlebitis, while secondary BCS is due to venous compression or invasion of an extrinsic lesion, such as a tumor, abscess, or cyst [4]. Antiphospholipid syndrome (APS) is an acquired cause of primary BCS.

Martens and Nevens reported that three common presenting features of BCS are abdominal pain (61%), ascites (83%), and hepatomegaly (67%), but the symptoms of BCS vary with its clinical presentation [5]. BCS is classified into four clinical types depending on the speed of onset, which are fulminant, acute, subacute, and chronic [6]. Fulminant BCS progresses rapidly (within days) and features hepatomegaly, abdominal pain, ascites, severe liver dysfunction with elevation of enzymes, hyperbilirubinemia, hepatic encephalopathy, clotting abnormality, and renal impairment. Acute BCS progresses within one month and clinical features are similar to fulminant case. Subacute BCS is the most common type and often progresses asymptomatically over approximately three months. Ascites or hepatic necrosis is mild and collateral circulation forms. Chronic BCS is characterized by portal hypertension and can be complicated by splenomegaly and esophageal varices. Histologically, congestive cirrhosis is observed. The subacute and chronic types are reported to account for 60% of BCS, while the fulminant and acute types are less frequent (5% and 20%, resp.) [7]. Espinosa et al. analyzed 43 patients with APS complicated by BCS and reported that 30 patients (70%) had subacute disease, 10 patients (23%) had chronic disease, and 3 patients (7%) had fulminant disease [2]. Thus, it is thought that acute BCS is rare in APS. Also, our patient had fever and evidence of marked inflammation, suggesting other causes, such as infection, so diagnosis of BCS was difficult.

Espinosa et al. [2] reported that 32 out of 43 patients had primary APS, 8 patients had APS related to systemic lupus erythematosus, and 3 patients had other types of APS. The average age of the patients was 30.8 years. BCS was the initial symptom of APS in 28 patients (65%), while 9 patients (21%) had a history of major venous occlusion. Symptoms included abdominal pain in 24 patients (56%), abdominal distension in 9 patients (21%), fever in 8 patients (19%), vomiting in 3 patients (7%), hepatomegaly in 25 patients (58%), ascites in 23 patients (53%), jaundice in 11 patients (26%), and splenomegaly in 4 patients (9%). Possible triggers of BCS were oral contraceptive medication, pregnancy, and major surgery. Lupus anticoagulant was positive in 77% and anticardiolipin antibody was positive in 94%.

For diagnosis of BCS, obstruction of hepatic venous outflow should be confirmed by invasive or noninvasive tests. Martens and Nevens reported that Doppler ultrasonography has a higher sensitivity and specificity for diagnosis of BCS among noninvasive tests [5]. Doppler ultrasonography findings in BCS include the following: (1) a large vein with absent, reversed, or turbulent flow; (2) large intrahepatic or subcapsular collaterals with continuous flow; (3) spider web appearance; (4) absent or flat hepatic vein waveform; and (5) a hyperechoic cord replacing a normal vein. In our patient, signs (1) and (3) were observed. She had a slight ascites and mild elevation of hepatic enzymes; we diagnosed acute BCS from clinical course and characteristic findings of abdominal ultrasonography. CT scan had previously revealed atrophy of the left lobe of the liver in this patient. Accordingly, when thrombotic occlusion of the middle hepatic vein and left hepatic vein blocked blood flow to the atrophic left lobe, elevations of liver enzymes were only mild.

Our patient had a history of pulmonary embolism/deep vein thrombosis, and she developed acute BCS despite anticoagulant therapy with warfarin. Castro et al. reported the APS patient who developed BCS during anticoagulant therapy [8]. However, Espinosa et al. reported that only 1 of 43 patients developed BCS during anticoagulant therapy [2], suggesting that it rarely occurs in APS patients who are on anticoagulants. Treatment of BCS includes thrombolytic therapy, percutaneous endovascular stenting, transjugular intrahepatic portosystemic shunting (TIPS), surgical shunting, and allogeneic liver transplantation. In Espinosa et al.'s report [2], the treatment to BSC patients of APS was anticoagulant therapy in 84% (anticoagulation alone in 50%), steroids in 37%, cyclophosphamide in 8%, aspirin in 11%, plasma exchange in 3%, surgery in 29%, and TIPS in 11%. Among the patients who received long-term anticoagulant therapy, the outcome was good in 76%. However, 6 of 31 patients of whom the outcome was known died of hepatic failure, sepsis, or bleeding due to thrombocytopenia.

Clinical characteristic of our patient was acute onset BSC with fever, acute abdomen, elevation of D-dimer, and CRP and was caused by APS. Acute BCS of APS was rarely reported. Although the disease is different, Karti et al. reported the acute BCS due to polycythemia vera patient who was treated with continuous heparin infusion combined with repeated phlebotomies and hydroxyurea [9]. And also our patient was suspected of having the complication of catastrophic APS because of highly inflammation state, but it was excluded since she was only impaired of the liver, not multiple organ failure. So she was treated with tight anticoagulant therapy with heparin sodium since the clinical feature of acute onset and the background of APS and succeeded to improve her abdominal pain and inflammation rapidly. In the case of organ dysfunction due to thrombosis in APS, especially catastrophic APS, there is no evidence to favor unfractionated heparin over low molecular weight heparin (LMWH) or warfarin; most critically ill patients should probably receive unfractionated heparin, given its reversibility [10]. However, according to clinical practical guidelines for vascular disease of the liver by European Association for the Study of Liver (EASL), the patient should be treated with LMWH for at least 5 to 7 days and also with oral anticoagulant treatment with vitamin K antagonist [11]. Heparin-induced thrombocytopenia was reported in BCS patients [12]. So, it is necessary to monitor the platelet count carefully when the patient with BSC caused by APS was treated with heparin.

Our patient had fever and evidence of marked inflammation. It was not ruled out etiologies such as infection, so that BCS was difficult to diagnose in our patient. But her symptoms responded to anticoagulant therapy alone; it is suggested that extensive thrombosis and vascular endothelial damage induced inflammatory reaction.

Our experience suggests that high-dose anticoagulant therapy can be effective in the early stage of BCS associated with APS before the onset of disseminated intravascular coagulation and multiple organ failure. If acute BCS is suspected in patients with APS, Doppler imaging and contrast-enhanced MRI of the hepatic/portal veins should be performed promptly and followed by early initiation of adequate anticoagulant therapy.
